# Analysis of the Microbial Community Structure of *Ixodes persulcatus* at Each Developmental Stage

**DOI:** 10.3390/ani15060830

**Published:** 2025-03-13

**Authors:** Yonghong Liu, Xiaonan Dong, Lianyang Sun, Hao Cui, Jiamei Kang, Nan Bu, Yishuai Zhang, Zehao Qi, Zixuan Li, Zilong Zhang, Li Zhao

**Affiliations:** 1 College of Veterinary Medicine, Inner Mongolia Agricultural University, Hohhot 010018, China; lyhdky@126.com (Y.L.); dxn1005@126.com (X.D.); 18748237178@163.com (L.S.); cuihao460009396@126.com (H.C.); kjm151476@163.com (J.K.); bunansyxy@163.com (N.B.); zyssyxy@163.com (Y.Z.); qzhsyxy@163.com (Z.Q.); lzxsyxy@163.com (Z.L.); 123zzl.z@163.com (Z.Z.); 2 Key Laboratory of Clinical Diagnosis and Treatment Technology in Animal Disease, Ministry of Agriculture and Rural Affairs, Hohhot 010010, China

**Keywords:** tick, *Ixodes persulcatus*, developmental stage, microbial population, high-throughput sequencing

## Abstract

**Simple Summary:**

In this study, we used *Ixodes persulcatus* at different developmental stages from Inner Mongolia as materials for nucleic acid extraction. Subsequently, we sequenced them on the Illumina NovaSeq 6000 platform. Illumina PE250 sequencing revealed that the seven developmental stages of *I. persulcatus* were annotated to 21 phyla, 43 classes, 104 orders, 188 families, 391 genera, and 556 species of bacteria. Among them, 4 phyla and 14 genera were present at all developmental stages, with Proteobacteria being the dominant phylum and *Rickettsia* spp. being the dominant genus. All developmental stages were annotated to a certain abundance of *Brucella* spp. Illumina PE150 sequencing revealed that the three samples of *I. persulcatus* were annotated to six orders, 28 families, 72 genera, and 158 species of viruses, of which 46 genera and 80 species were found in all three sample species. To the best of our knowledge, this is the first study that comprehensively analyzed the microbial community composition of *I. persulcatus* at different developmental stages. Based on the study outcomes, certain abundance of *Rickettsia japonica*, bovine viral diarrhea virus, and African swine fever virus were annotated to *I. persulcatus*.

**Abstract:**

Ticks are the second most significant vector of pathogens worldwide. *Ixodes persulcatus* is one of the dominant tick species in Inner Mongolia that can carry and transmit various pathogenic microorganisms. However, only one specific pathogen has been detected in a particular developmental stage of *I. persulcatus*, moreover metagenomic analysis has been conducted only in the adult tick stage. In this study, we used *I. persulcatus* at different developmental stages (first-generation female adult ticks, eggs, larval ticks, engorged larval ticks, nymphal ticks, engorged nymphal ticks, and second-generation adult ticks) from Inner Mongolia as materials for nucleic acid extraction. Subsequently, we constructed Illumina PE250 and Illumina PE150 libraries and sequenced them on the Illumina NovaSeq 6000 platform. Finally, we used molecular biology software and sequence analysis platform to analyze microbial community structures. Illumina PE250 sequencing revealed that the seven developmental stages of *I. persulcatus* were annotated to 21 phyla, 43 classes, 104 orders, 188 families, 391 genera, and 556 species of bacteria. Among them, 4 phyla and 14 genera were present at all developmental stages, with Proteobacteria being the dominant phylum and *Rickettsia* spp. being the dominant genus. In addition, *Rickettsia* had the highest relative abundance in the seven developmental stages. All developmental stages were annotated to a certain abundance of *Brucella* spp. Illumina PE150 sequencing revealed that the three samples (X-I-YDCP: first-generation adult ticks; X-I-MIX: mixed samples of eggs, larval ticks, and nymphal ticks; X-I-EDCP: second-generation adult ticks) of *I. persulcatus* were annotated to six orders, 28 families, 72 genera, and 158 species of viruses, of which 46 genera and 80 species were found in all three sample species. To the best of our knowledge, this is the first study that comprehensively analyzed the microbial community composition of *I. persulcatus* at different developmental stages. Based on the study outcomes, certain abundance of *Rickettsia japonica*, bovine viral diarrhea virus, and African swine fever virus were annotated to *I. persulcatus*.

## 1. Introduction

*Ixodes persulcatus* (*I. persulcatus*) is one of the dominant tick species in Inner Mongolia, China [1]. *I. persulcatus* is a typical three-host tick [2], and its life cycle includes four developmental stages: egg, larva, nymph, and adult [3]. *I. persulcatus* was feed on 46 species of hosts, which belong to 22 families [4]. *I. persulcatus* can cause direct harm to the host by biting and sucking blood [5]. More importantly, this species can carry and transmit multiple pathogenic microorganisms. For example, *Borrelia miyamotoi* was first discovered in adult *I. persulcatus* ticks in Japan [6]. Tick-borne encephalitis virus (TBEV) was identified in adult *I. persulcatus* in the Chelyabinsk Region in Russia [7]. *Anaplasma phagocytophilum*, *Ehrlichia muris*, *Borrelia miyamotoi*, *Borrelia afzelii*, *Borrelia garinii*, *Babesia canis*, *Babesia microti*, *Babesia venatorum*, *Babesia divergens*, and *Theileria cervi* were also detected in adult ticks in many regions in Russia [8]. *I. persulcatus* is also the main vector of *Borrelia*, *Anaplasma*, and *Babesia* infections in Mongolia [9]. In addition, *B*. *miyamotoi*, *B*. *afzelii*, *B*. *garinii*, and *Borrelia burgdorferi* have been detected in unfed nymphal and adult ticks in Finland [10], whereas *B*. *afzelii*, *B*. *garinii*, and *Borrelia valaisiana* were detected in nymphal and adult ticks in Sweden [11]. In China, Liu et al. [12] conducted metagenomic analysis to annotate 17 viruses from 9 genera in *I. persulcatus* adult ticks in Heilongjiang and Jilin, which included Tahe rhabdovirus 2, Tahe rhabdovirus 3, Beiji nairovirus, Yichun nairovirus, Mukawa virus, Mudanjiang phlebovirus, Sara tick phlebovirus, Onega tick phlebovirus, Nuomin virus, Yichun mivirus, TBEV, Alongshan virus, Jilin partiti-like virus 1, Yichun tombus-like virus, Jilin luteo-like virus 2, *Ixodes scapularis* associated virus, and *I. scapularis* associated virus 3. In Inner Mongolia, adult ticks were annotated to 594 genera; using nested PCR detected *Candidatus Rickettsia tarasevichiae*, *B. garinii*, *B. afzelii*, and *B. miyamotoi* [13]. In addition, Alongshan virus was found in *I. persulcatus* in Inner Mongolia and Heilongjiang [14], whereas YEZO virus [15], TBEV [16], *B. burgdorferi* [17], and spotted fever group *Rickettsia* [18] were detected in Inner Mongolia, Heilongjiang, and Jilin. In addition, 32 species of bacteria and viruses were detected in China [19]. The above reports are sufficient to demonstrate that *I. persulcatus* has a latent ability to carry pathogenic microorganisms. Considering the different types of pathogens carried and transmitted by ticks across different geographical distributions [20], it is necessary to comprehensively analyze the microbial diversity carried by *I. persulcatus* in Inner Mongolia.

High-throughput sequencing can rapidly and efficiently analyze microbial communities without the need for bacterial culture; moreover, it can identify uncultured bacteria and unknown pathogens [12,20,21]. Previous studies on *I. persulcatus* have only focused on the detection of specific pathogens carried at a certain developmental stage or metagenomic analysis of adult tick stage. In this study, we conducted high-throughput sequencing of *I. persulcatus* at different developmental stages under laboratory feeding conditions using 16S rDNA amplicons and viral metagenomics techniques to determine their microbial community structure of *I. persulcatus* at different developmental stages and provide a scientific basis for the prevention and control of *I. persulcatus* and its transmitted diseases.

## 2. Materials and Methods

### 2.1. Sample Collection

**Figure 1:**
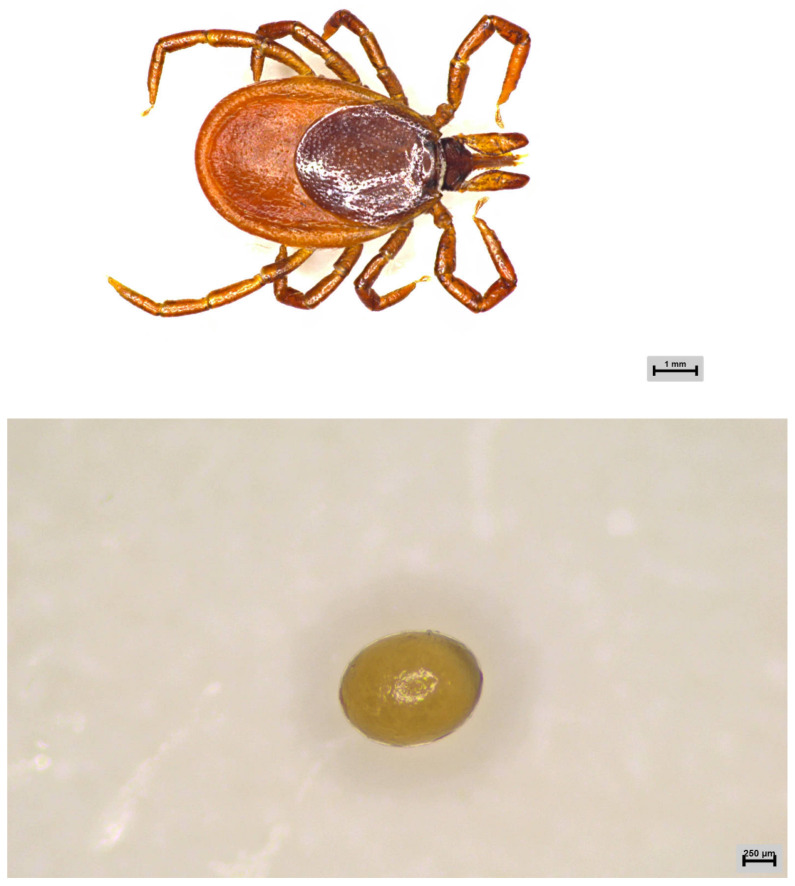

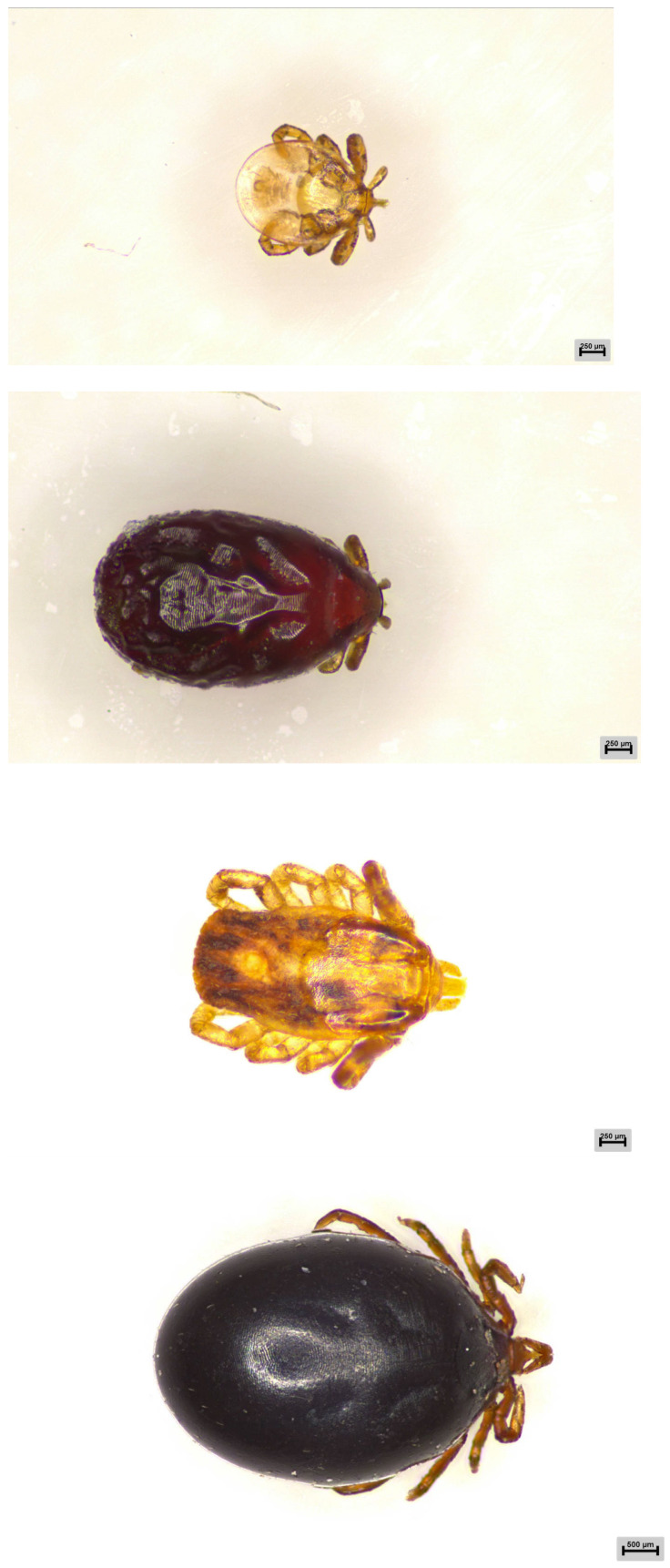

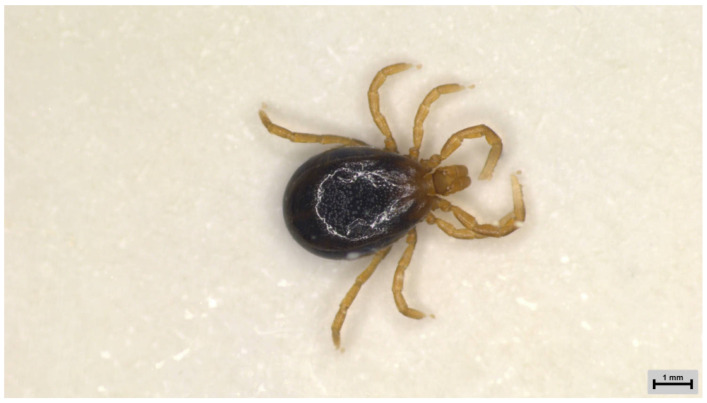
*Ixodes persulcatus* at the different developmental stages examined in this study (1-1: first-generation female adult ticks; 1-2: eggs; 1-3: larval ticks; 1-4: engorged larval ticks; 1-5: nymphal ticks; 1-6: engorged nymphal ticks; and 1-7: second-generation adult ticks).

First-generation engorged female adult ticks and male adult ticks were collected from cattle in Hinggan League in the Inner Mongolia Autonomous Region, China (950 m above sea level; 46°77′ N, 120°30′ E). In the laboratory, after oviposition, male and female adult ticks were identified as *I. persulcatus* through morphological and molecular biological identification methods. The remaining six developmental stages of *I. persulcatus* (X-I-PL: eggs; X-I-YP: larval ticks; X-I-BXYP: engorged larval ticks; X-I-RP: nymphal ticks; X-I-BXRP: engorged nymphal ticks; and X-I-EDCP: second-generation adult ticks) were obtained from the first-generation female adult ticks (X-I-YDCP) collected from the field and artificially raised in the laboratory (Figure 1); the samples were stored at −80 °C.

### 2.2. DNA and RNA Extraction

Each sample represented a pool of ticks in the same developmental stage. The surface of the samples was washed with sterile water and 75% ethanol. DNA was extracted using TaKaRa Mini BEST Universal Genomic DNA Extraction Kit Ver. 5.0 (Takara, Beijing, China) from seven developmental stage samples of *I. persulcatus*. RNA was extracted using RaPure Viral RNA/DNA Kit (MAGEN, Shanghai, China) from three mixed samples of *I. persulcatus* (X-I-YDCP: first-generation adult ticks; X-I-MIX: mixed samples of eggs, larval ticks, and nymphal ticks; and X-I-EDCP: second-generation adult ticks) using RaPure Viral RNA/DNA Kit (MAGEN, Shanghai, China). Thereafter, nucleic acid concentration was determined, and samples were stored at −80 °C.

### 2.3. Library Construction and High-Throughput Sequencing

The DNA extracted from each sample was used as a template to amplify the 16S rDNA V3-V4 variable region using TransStart R^©^ FastPfu DNA polymerase (TransStart, Beijing, China). The universal primers 338F (5′-ACTCCTACGGGAGGCAGCA-3′) and 806R (5′- GGACTACHVGGGTWTCTAAT-3′) were used under the following conditions: 94 °C for 3 min; 30 cycles of 94 °C for 30 s, 56 °C for 30 s, and 72 °C for 45 s; and a final cycle of 72 °C for 5 min. The PCR products were recovered using AxyPrep DNA Gel Extraction Kit (Axygen, Suzhou, China), followed by the Illumina PE250 library was constructed using VAHTS^®^ ssDNA Library Prep Kit (Illumina, San Diego, CA, USA). High-throughput 16S rRNA sequencing was performed on the Illumina NovaSeq 6000 platform (San Diego, CA, USA).

After RNA fragmentation of each sample, 8 μL of the First Strand-Buffer was mixed with first-strand enzyme, and PCR was performed for synthesizing cDNA, SEAEnzyme MIX, and SEA Buffer were added for end-repair and A-addition reaction. Finally, the Illumina PE150 library was constructed using Fast RNA-Seq Library Prep Kit (Illumina, San Diego, USA), and viral metagenomics sequencing was performed on the Illumina NovaSeq 6000 platform (San Diego, CA, USA).

### 2.4. Data Analysis

According to the barcode, we first obtained the Illumina PE250 sequencing sequence. Then, the FLASH software (V.1.2.11) was used to merge the paired-end reads of the original DNA fragments. Subsequently, these sequences were subjected to quality inspection, filtering, and assembled. Usearch (V.7.0.1090, http://drive5.com/uparse/) (accessed on 20 April 2024) was used for sequence analysis. The Qiime platform (V1.9.0, http://qiime.org/scripts/assign_taxonomy.html) (accessed on 20 April 2024) and the RDP Classifier (V.2.2, http://sourceforge.net/projects/rdp-classifier/) (accessed on 20 April 2024) were used to classify the sequences with ≥97% similarity and generate operational taxonomic units (OTUs). The Bayesian algorithm was used to classify and analyze the OTUs.

Next, we analyzed the alpha diversity of the samples by the mothur website (V.1.41.1, http://www.mothur.org/wiki/Schloss_SOP#Alpha_diversity) (accessed on 20 April 2024). The alpha diversity included the number of unique OTUs for each sample, the Chao index (http://www.mothur.org/wiki/Chao) (accessed on 20 April 2024) of community richness, Shannon index (http://www.mothur.org/wiki/Shannon) (accessed on 20 April 2024) of community diversity, the Simpson index (http://www.mothur.org/wiki/Simpson) (accessed on 20 April 2024), and the Good’s coverage (http://www.mothur.org/wiki/Coverage) (accessed on 20 April 2024) of sequencing depth index. These parameters combined with species accumulation curves were used to determine the sample community diversity and predict species richness. Finally, the results were visualized using Venn diagrams and histograms.

For the Illumina PE150 sequencing sequence, we first used Cutadapt (V.1.16, https://cutadapt.readthedocs.io/en/stable/) (accessed on 20 April 2024) and FastQC (V.0.11.4, https://www.bioinformatics.babraham.ac.uk/projects/fastqc/) (accessed on 20 April 2024) for optimization and quality control. Then, we used megahit (V.1.2.9, https://github.com/voutcn/megahit) (accessed on 20 April 2024) and MetaGeneMark (V.3.38, http://exon.gatech.edu/GeneMark/) (accessed on 20 April 2024) to splice and assemble high-quality sequences and predict genes. Finally, we used diamond (https://github.com/bbuchfink/diamond) (accessed on 20 April 2024) to annotate the obtained genes. Based on the above analysis, we visualized the results in Venn diagrams and histograms after similar clustering, group sorting, and difference comparison.

Sequencing and sequence analysis were performed in collaboration with Origin-gene biology Co., Ltd. (Shanghai, China).

## 3. Result

### 3.1. Sequencing Results of Illumina PE250

#### 3.1.1. General Statistics

The sequencing results of the seven samples were corrected, and chimeras were removed to obtain 1,329,737 optimized sequences, including 193,013 from first-generation female adult ticks, 77,459 from eggs, 691,824 from larval ticks, 141,628 from engorged larval ticks, 78,104 from nymphal ticks, 54,276 from engorged nymphal ticks, and 93,433 from second-generation adult ticks. The total base number of the optimized sequences was 551,122,016 bp, and the average length of the sequences was 414.46 bp (Table 1).

#### 3.1.2. Alpha Diversity Analysis

**Figure 2:**
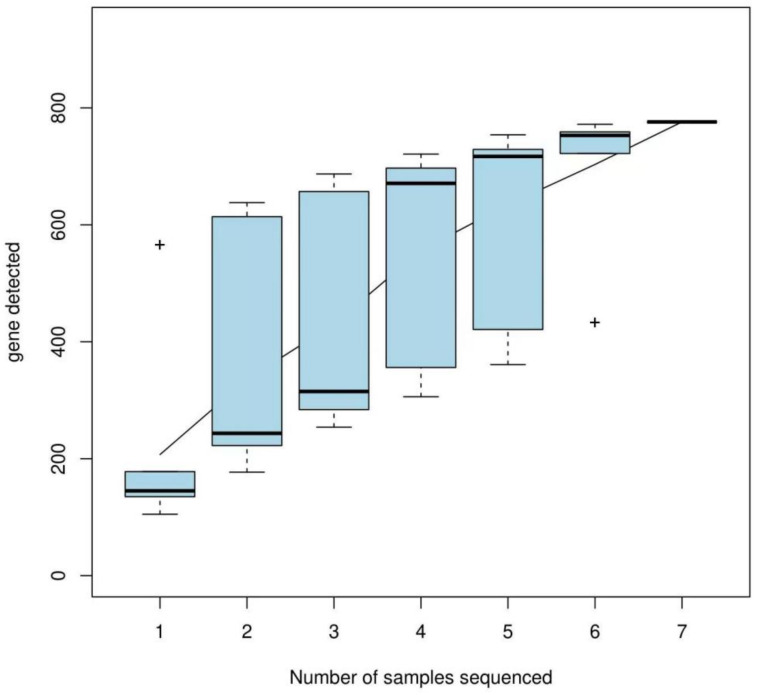
Species accumulation curves (the horizontal axis indicates the sample size and the vertical axis indicates the number of OTUs after sampling).

The alpha diversity index was calculated based on a 97% similarity threshold. The Shannon index revealed the following trend: engorged larval ticks > second-generation adult ticks > nymphal ticks = larval ticks > first-generation female adult ticks > eggs > engorged nymphal ticks. The Simpson index revealed the following trend: engorged nymphal ticks > eggs > first-generation female adult ticks > larval ticks > engorged larval ticks > nymphal ticks > second-generation adult ticks. The Chao index and abundance-based coverage estimator (ACE) index revealed the following trend: engorged larval ticks > first-generation female adult ticks > larval ticks > second-generation adult ticks > eggs > engorged nymphal ticks > nymphal ticks. For all samples, the Good’s coverage was >99.9% (Table 2). The species accumulation curves (Figure 2) demonstrated that the OTU index increased linearly with an increase in sample size.

#### 3.1.3. OTU Cluster Analysis

**Figure 3:**
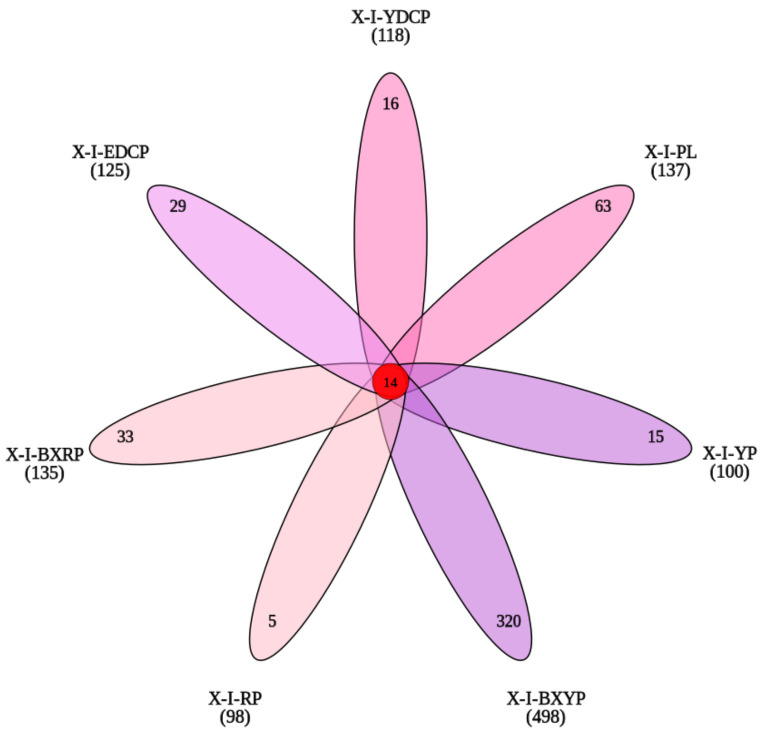
Venn diagram of the seven samples based on OTUs (X-I-YDCP: first-generation female adult ticks; X-I-PL: eggs; X-I-YP: larval ticks; X-I-BXYP: engorged larval ticks; X-I-RP: nymphal ticks; X-I-BXRP: engorged nymphal ticks; and X-I-EDCP: second-generation adult ticks).

First-generation female adult ticks, eggs, larval ticks, engorged larval ticks, nymphal ticks, engorged nymphal ticks, and second-generation adult ticks obtained 118, 137, 100, 498, 98, 135, and 125 OTUs, respectively. *I. persulcatus* had 14 high-similarity OTUs at all developmental stages, with 16 (13.55%, 16/118), 63 (45.99%, 63/137), 15 (15.00%, 15/100), 320 (64.26%, 320/498), 5 (5.10%, 5/98), 33 (24.44%, 33/135), and 29 (23.20%, 29/125) unique OTUs in the seven developmental stages (Figure 3). In all samples, more than 91.97% of the tags were assigned at the genus level, and only some tags could be classified at the species level.

#### 3.1.4. Microbial Population

##### Microbial Community Composition at the Phylum Level

**Figure 4:**
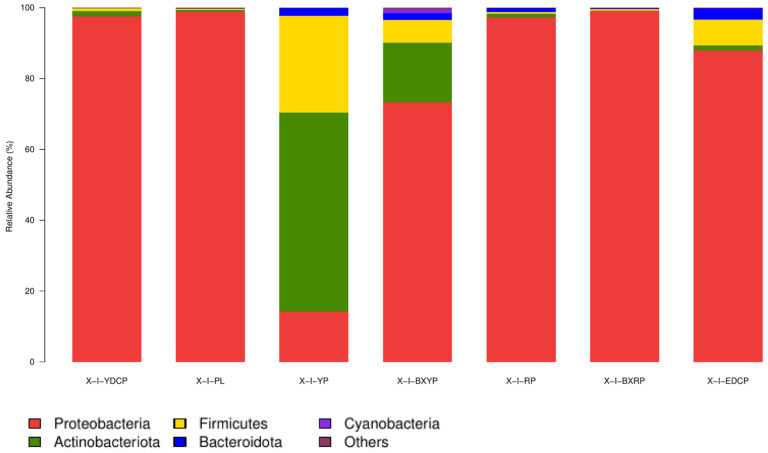
Microbial community composition at the phylum level (To improve the image, the section with a relative abundance of <1% has been merged into the other section and displayed in the graph).

For *I. persulcatus*, 21 bacterial phyla were identified, among which 4 (Proteobacteria, Actinobacteria, Firmicutes, and Bacteroidota) were common to all seven developmental stages, and the total abundances were >1%. Proteobacteria had the highest total abundance (81.13%) in all samples, and its relative abundance in the seven developmental stages ranged from 14.19% to 99.01%, showing a marked predominance. Actinobacteria (11.08%) had the second highest total abundance, and its relative abundance was the highest in larval ticks (56.23%), followed by engorged larval ticks (16.78%). However, its relative abundance was very low in eggs (0.43%) and engorged nymphal ticks (0.08%). The total abundance of Firmicutes in all samples was 6.18%, and its relative abundance in each developmental stage ranged from 0.42% (eggs) to 27.30% (larval stage). The total abundances of Bacteroidetes in all samples was 1.31%, and its relative abundance in each developmental stage ranged from 0.15% to 3.22%. The relative abundances of Cyanobacteria in the engorged larval ticks (1.13%) stage was >1% (Table 3, Figure 4).

##### Microbial Community Composition at the Genus Level

**Figure 5:**
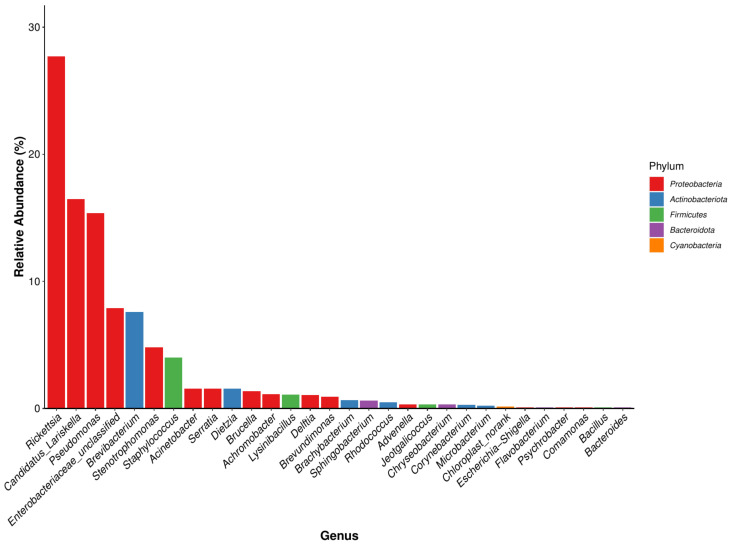
Histogram of the dominant genus of *I. persulcatus* (Bacterial genus in the same color indicates that they are derived from the same bacterial phylum).

**Figure 6:**
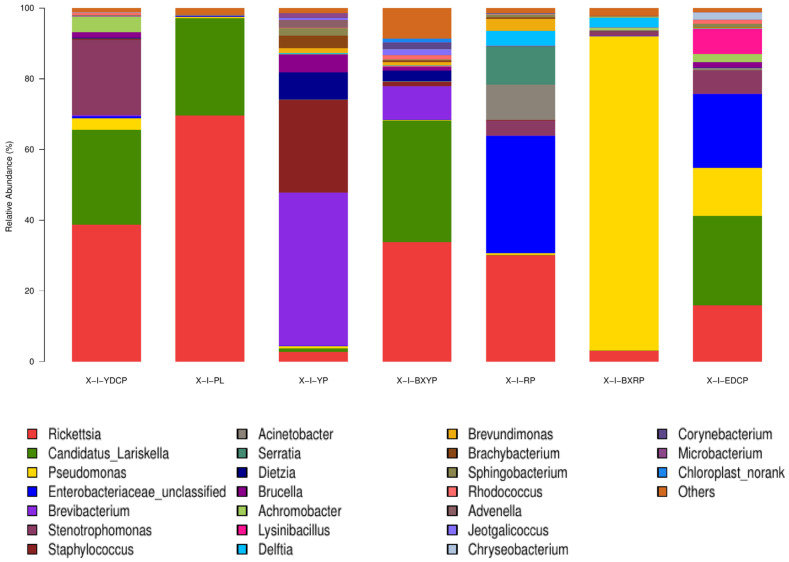
Microbial community composition at the genus level of seven samples (Note: To improve the image, the section with a relative abundance of <1% has been merged into the other section and displayed in the graph).

In total, 391 bacterial genera were identified, of which 14 genera were common in all seven developmental stages. Among the 30 genera with a high total abundance (a total abundance of more than 1%), 15 genera belonged to Proteobacteria, and the top 4 bacterial genera were all Proteobacteria (Figure 5). The bacterial genus with the highest total abundance was *Rickettsia* (27.72%), and its relative abundance at different developmental stages of *I. persulcatus* varied considerably. The developmental stages that showed the highest and lowest relative abundances of *Rickettsia* were eggs (69.596%) and larval ticks (2.714%), respectively. *Rickettsia* was also the bacterial genus with the highest relative abundance in first-generation female adult ticks. The total abundance of *Candidatus Lariskella* (16.459%) was second only to that of *Rickettsia*, and it ranged from 34.363% (engorged larval ticks) to 0.034% (engorged nymphal ticks). *Candidatus Lariskella* was also the bacterial genera with the highest relative abundance in the engorged larval ticks and the second-generation adult ticks. Pseudomonas spp. was mainly found in the engorged nymphal ticks (88.883%), whereas its relative abundance in the engorged larval ticks was low (0.259%). Unclassified *Enterobacteriaceae* showed the highest relative abundance in nymphal ticks (33.252%) but was only 0.006% in larval ticks. *Brevibacterium* was the genera with the highest relative abundance in the larval ticks (43.261%), but its abundance in engorged larval ticks (9.384%) was significantly lower. Its abundance was also low in the nymphal ticks (0.038%), engorged nymphal ticks (0.011%), first-generation female adults (0.304%), and second-generation adults (0.030%), and it was not annotated at all in the eggs. In addition, some common bacterial genera were found, including *Staphylococcus*, *Escherichia*, *Shigella*, and *Brucella* (Table 4). In summary, the dominant genera detected in *I. persulcatus* at different developmental stages were different (Figure 6). There were also differences in the overall dominant genera of the samples (Figure 5), and some bacterial genera existed only at specific developmental stages.

##### Microbial Community Composition at the Species Level

**Figure 7:**
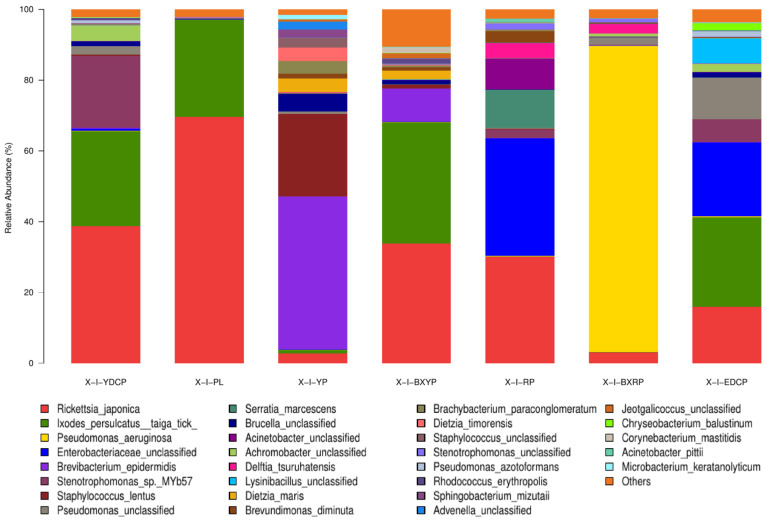
Microbial community composition at the species level (Note: To improve the image, the section with a relative abundance of <1% has been merged into the other section and displayed in the graph).

In total, 556 bacterial species were annotated to the samples of *I. persulcatus* at different developmental stages, and the species with total abundances of >1% were *Rickettsia japonica*, *I*. *persulcatus taiga tick*, *Pseudomonas aeruginosa*, unclassified *Enterobacteriaceae*, *Brevibacterium epidermidis*, *Stenotrophomonas* sp. MYb57, *Staphylococcus lentus*, unclassified *Pseudomonas*, *Serratia marcescens*, unclassified *Brucella* spp., unclassified *Acinetobacter*, unclassified *Achromobacter*, *Delftia tsuruhatensis*, and unclassified *Lysinibacillus* (Table 5). The abundances of these bacteria varied at the different developmental stages of *I. persulcatus* (Figure 7).

### 3.2. Sequencing Results of Illumina PE150

#### 3.2.1. Species Classification Annotation Data

Illumina PE150 sequencing of three samples of *I. persulcatus* revealed a total of 166,029,882 reads (first-generation adult ticks: 83,476,626 reads, mixed sample: 18,763,724 reads, second-generation adult ticks: 63,789,532 reads). After quality control, the raw data contained 165,842,692 reads (83,331,358, 18,734,556, and 63,776,778, respectively). The contents of CG % were 53.32%, 53.68%, and 54.59%, respectively. After open reading frame (ORF) prediction, 1,726,458 reads (2.0718%, 1,726,458/83,331,358), 114,072 reads (0.6089%, 114,072/18,734,556), and 2,608,782 reads (4.0905%, 2,608,782/63,776,778), respectively, were annotated as viruses.

#### 3.2.2. Species Composition at the Order Level

**Figure 8:**
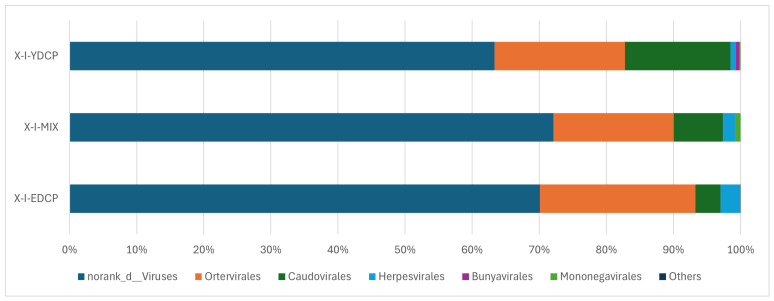
Species composition distribution at the order level (To improve the image, the section with a relative abundance of <1% has been merged into the other section and displayed in the graph).

The three samples were annotated to six virus orders (Table 6). Among them, four virus orders coexisted in all three samples, and the order of their abundance from high to low was norank d Viruses, Ortervirales, Caudovirales, and Herpesvirales. In addition, Bunyavirales with low abundance was annotated to first-generation adult ticks and second-generation adult ticks, and Mononegavirales was annotated to first-generation adult ticks and the mixed sample (Figure 8).

#### 3.2.3. Species Composition at the Family Level

**Figure 9:**
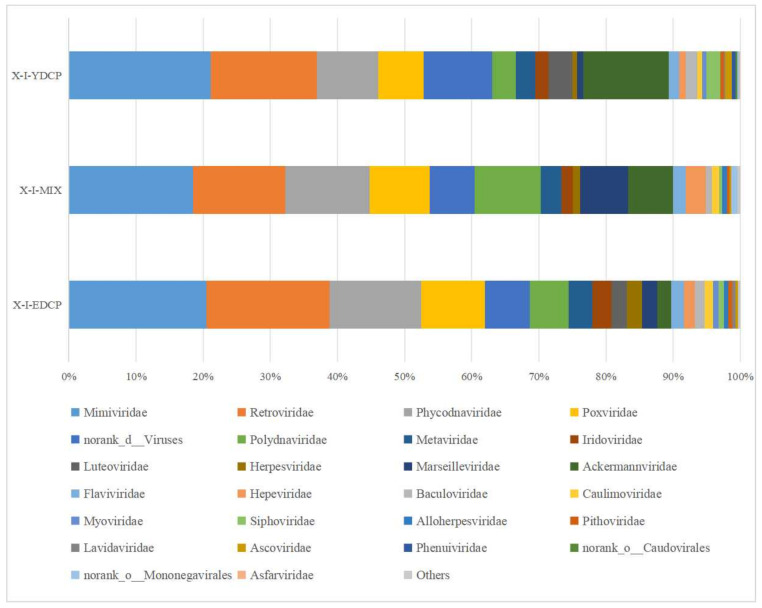
Species composition distribution at the family level (Note: To improve the image, the section with a relative abundance of <1% has been merged into the other section and displayed in the graph).

Overall, three samples were annotated to 28 viral families and coannotated to 21 families, such as *Mimiviridae*, *Retroviridae*, *Phycodnaviridae*, *Poxviridae*, and *Ackermannviridae*. Notably, the first-generation and second-generation adult ticks were also coannotated to five families, namely, *Luteoviridae*, *Phenuiviridae*, norank o *Caudovirales*, *Asfarviridae*, and *Nimaviridae*. The first-generation adult ticks and the mixed sample were also coannotated to norank o Mononegavirales. Meanwhile, mixed sample and second-generation adult ticks were also coannotated to Hepadnaviridae (Figure 9).

#### 3.2.4. Species Composition at the Genus Level

**Figure 10:**
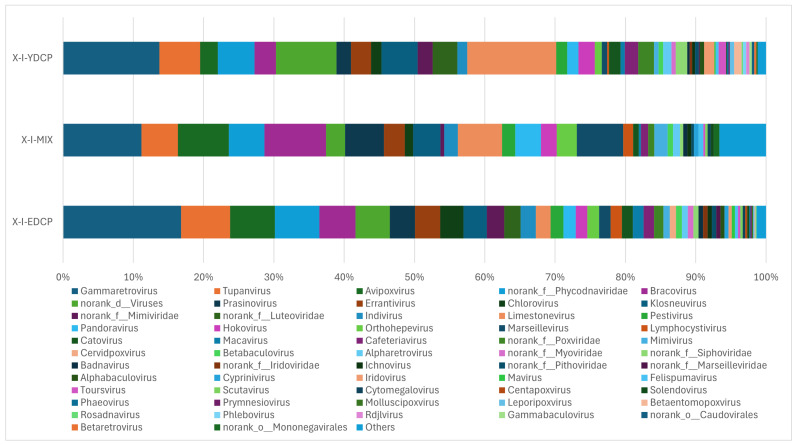
Species composition distribution at the genus level (Note: To improve the image, the section with a relative abundance of <1% has been merged into the other section and displayed in the graph).

The three samples were annotated to 72 genera, the first-generation adult ticks were annotated to 64 genera, mixed samples were annotated to 51 genera, and second-generation adult ticks were annotated to 71 genera, of which 46 genera were found in all three samples. The total abundance of *Gammaretrovirus* was the highest of all three samples. First-generation and second-generation adult ticks were also coannotated to 17 genera. First-generation adult ticks and mixed sample were also coannotated to norank o *Mononegavirales*. Second-generation adult ticks and mixed sample were also coannotated to 50 genera. In addition, second-generation adult ticks were annotated to four genera (V5virus, *Hepacivirus*, *Rhadinovirus*, and *Batrachovirus*) that were unique to this stage (Figure 10).

#### 3.2.5. Species Composition at the Species Level

**Figure 11:**
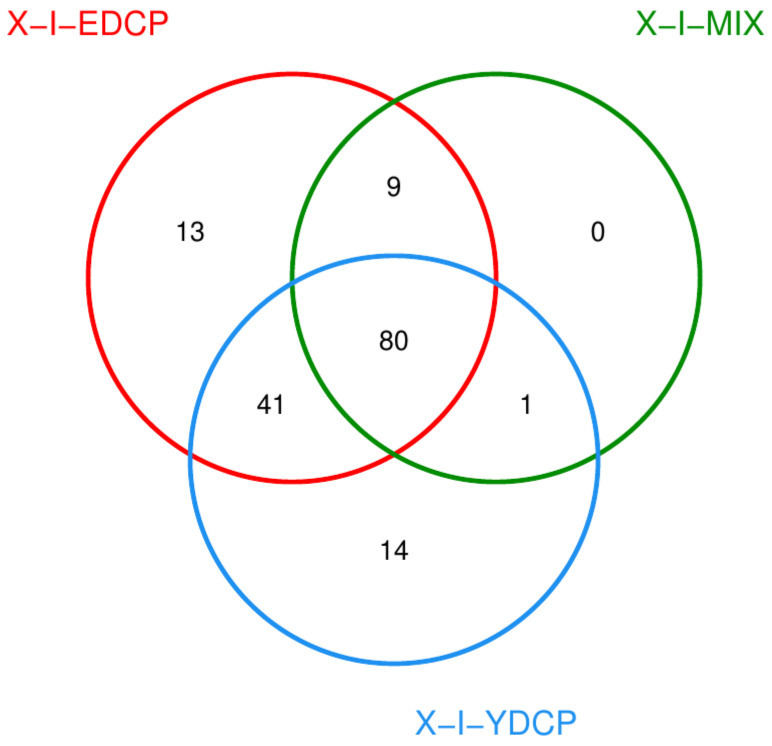
Venn diagram of species (X-I-YDCP: first-generation adult ticks; X-I-MIX: mixed sample; X-I-EDCP: second-generation adult ticks).

**Figure 12:**
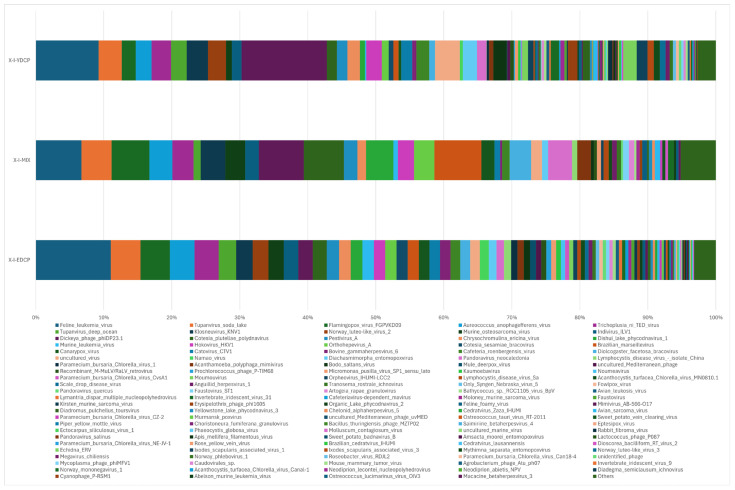
Species composition distribution at the species level (To improve the image, the section with a relative abundance of <1% has been merged into the other section and displayed in the graph).

Overall, three samples were annotated to 158 species, of which 80 species were jointly annotated to all three samples (Figure 11). Among them, *feline leukemia virus*, *Pestivirus A*, *Flamingopox virus* FGPVKD09, *Sheeppox virus*, *Diachasmimorpha entomopoxvirus*, *Mythimna separata entomopoxvirus*, *Amsacta moorei entomopoxvirus*, *Canarypox virus*, *Murmansk poxvirus*, *Fowlpox virus*, *Mule deerpox virus,* and *Eptesipox virus* were notable. First-generation adult ticks and second-generation adult ticks were coannotated to 41 species, including African swine fever virus (ASFV). First-generation adult ticks and mixed sample were also coannotated to Norway mononegavirus 1, and second-generation adult ticks and mixed sample were also coannotated to nine species. In addition, first-generation adult ticks were annotated to 14 species unique species, second-generation adult ticks were annotated to 13 species unique species, and mixed samples were not annotated to any unique species (Figure 12).

## 4. Discussion

From 1950 to 2018, 124 tick species, 103 tick-borne agents, and 29 tick-borne pathogens were reported in China [19,20]. Meanwhile, new tick-borne pathogens are emerging [14,15,22]. The distribution of *I. persulcatus* ranges from the Baltic Sea to the Pacific Ocean, and *I. persulcatus* is mainly found in Eurasia [23]. *I. persulcatus* is also one of the dominant tick species in Inner Mongolia, China [1]. *I. persulcatus* can carry a variety of pathogens and cause various infectious diseases [18]. However, only a few studies have investigated *I. persulcatus*’s ability to carry and transmit pathogens, and most previous studies have focused on the detection of specific microorganism carried by *I. persulcatus* only at a certain developmental stage [6,7,8,9,10,11,14,15,16,17,18,20] or metagenomic analysis of adult tick stage [12,13]. In the present study, we used high-throughout sequencing to analyze the microbial community structures of *I. persulcatus* in Inner Mongolia at various developmental stages.

Regarding the high-throughput sequencing results of the 16S rRNA V3–V4 region, a larger the Shannon index and a smaller Simpson index indicated a higher microbial community diversity of the sample. The larger the ACE value, the greater the total number of species [20,24,25]. The results revealed that the diversity of bacteria was the lowest in the engorged nymphal ticks, and the total number of species was the highest in the engorged larval ticks. The sequencing depth index Good’s coverage, species accumulation curves, and OTU clustering analysis of *I. persulcatus* samples indicate that the sequencing depth is sufficient to reflect the microbial diversity of the sample. This can reflect the microbial community’s richness of the *I. persulcatus*, and some microbial populations are the same among the seven developmental stages of *I. persulcatus*. Based on the estimators, the sequencing data in this study showed that Proteobacteria was the dominant phylum in *I. persulcatus* in Inner Mongolia, which was consistent with the dominant phylum in other ticks, such as *Dermacentor nuttalli* [20], *I. scapularis* [26], *Dermacentor occidentalis* [27], *Haemaphysalis longicornis* [28], *Hyalomma scupense* [29], *Haemaphysalis flava* [24], *Amblyomma maculatum* [30], and *Amblyomma tuberculatum* [31]. This similarity may be attributed to the wide distribution of Proteobacteria in nature, revealing that Proteobacteria have strong adaptability and can survive in different tick species.

The dominant genus in *I. persulcatus* was *Rickettsia*, which was annotated at all developmental stages; however, the reason for the large difference in relative abundances among different developmental stages remains unknown. It has been reported that blood-feeding behaviors alter the composition and abundance of insect microbiomes [32]. For example, the community diversity of *Anopheles gambiae* decreased significantly after blood meals [33], midgut microbial richness of *Aedes aegypti* increased after blood feeding [34], and the midgut microbial richness of sand flies decreased after blood feeding [35,36]. In *Melophagus ovinus*, the relative abundances of *Bartonella* increased, whereas those of *Wolbachia* and most other bacterial genera decreased after blood feeding [25]. It has also been reported that the molting process of ticks causes the discharge of a large amount of obligate, intracellular, parasitic bacterium [37]. *Rickettsia* spp. is an obligate, intracellular, parasitic genus that is mainly transmitted via arthropods [24]. Certain *Rickettsia* species can be transmitted to the host through the salivary glands during bites [38,39]. In this study, all seven developmental stages included *Rickettsia japonica*, the causative agent of Japanese spotted fever (JSF). The main symptoms of JSF are fever, erythema, and eschar [40]. In severe cases, JSF can lead to inflammation of the central nervous system and even death [41,42]. Previous reports on *R. japonica* have mainly focused on hosts such as *Ha. longicornis*, *Haemaphysalis hystricis*, *Ha. flava*, and *Rhipicephalus microplus* [19]. Therefore, *I. persulcatus* may be a newly discovered potential transmission host of *R. japonica*.

*Brucella* spp. was annotated in all seven developmental stages of *I. persulcatus* in this study. It has been reported that at least 16 species of ticks can carry *Brucella* spp. [43]. *Brucella* has also been shown to exist at all life stages of ticks [44]; moreover, *Brucella* spp. can be transmitted vertically in ticks [45,46]. Recent studies have shown that *Brucella* spp. can be detected in various tissues and organs of ticks [43] and can cause Brucellosis, which is widespread in more than 170 countries across five continents worldwide [47]. Brucellosis is an animal disease that must be reported by the World Organization for Animal Health. It is one of the most important neglected zoonotic diseases recognized by many international organizations [20]. Inner Mongolia is one of the most seriously affected areas by *Brucella* infection. The seroprevalence of brucellosis in cattle in Inner Mongolia is the second highest in China [48]. *Brucella* also exists in another dominant tick species in Inner Mongolia—*D*. *nuttalli* [20], which exhibits transovarial transmission potential [49]. This also reveals the potential risk of *Brucella* spp. transmission through ticks in Inner Mongolia.

Notably, due to the large population, wide distribution, and strong environmental adaptability of ticks in nature, tick-borne viruses can exist in nature for a long periods and even undergo mutations, thereby increasing the difficulty of preventing and controlling tick-borne virus diseases [50]. In this study, three samples were annotated to six orders, 28 families, 72 genera, and 158 species of virus. In particular, *Pestivirus* A (bovine viral diarrhea virus 1) was annotated with considerable abundance in all three samples. Bovine viral diarrhea virus (BVDV) belongs to *Flaviviridae*, *Pestivirus* [51]. BVDV infection leads to persistent infection and immunosuppression in cattle, and the virus can also adopt some immune evasion strategies to help itself replicate successfully [52]. This may indicate the importance of *I. persulcatus* in BVDV prevention and control. This study also annotated ASFV with low abundance in first-generation adult ticks and second-generation adult ticks. ASFV is an acute, highly lethal infectious porcine virus with a 100% mortality rate [53]. In 2018, ASFV was first reported in Liaoning Province, China [54], subsequently spread to all provinces in the mainland China [55], and impacting the global pork supply [56]. ASFV can proliferate in ticks [56], transmit transstadially and sexually [57,58], and cause transovarial infection [59]. This reveals that ticks can be the biological carriers of ASFV. It has been confirmed that eight species of soft ticks can transmit ASFV [60]. Meanwhile, a DNA fragment of ASFV has been detected in *Dermacentor silvarum*, *D*. *nuttalli*, *Dermacentor niveus* [61], *Dermacentor reticulatus*, and *Ixodes ricinus* [62]. However, there is no evidence that these hard ticks can transmit ASFV [63]. Further studies are required to determine whether *I. persulcatus* is a biological or mechanical vector of ASFV.

The microbial community composition and diversity of ticks are affected by several factors, including tick species and host [32,64], sex [65], developmental stage [66], blood meal [67], engorgement [68,69], and geographical location [70]. Microorganisms that colonize ticks include endosymbionts, potentially pathogenic pathogens, and nonpathogenic organisms [12]. Endosymbionts have symbiotic, reciprocal, or parasitic relationships with ticks and play important roles in their entire life cycle [71]. Ticks play an important role in microbial survival and transmission, modulating reproductive fitness, vector competence, and nutrition supply. Studying the microorganisms of ticks can further aid in the understanding of the interaction between microorganisms and vectors of ticks as well as in the development of biological control tools for ticks [12]. In this study, we reported the dynamic changes and richness of microorganisms in different developmental stages of *I. persulcatus*. However, this report may be the tip of the iceberg in terms of the actual number of pathogens carried and transmitted by *I. persulcatus*. Tick-borne pathogens that infect livestock may eventually lead to human disease. Therefore, future studies should confirm the pathogens carried by *I. persulcatus* and the pathogenicity of these pathogens. In summary, this study revealed the microorganisms in *I. persulcatus* and expanded the spectrum of pathogens that may exist in *I. persulcatus.* This study has medical and veterinary significance and can aid in the prediction emerging pathogens, assessment of the potential risks of animal and public health, development of biological control tools, and the prevention of insect-borne diseases.

## 5. Conclusions

In this study, we annotated 21 phyla, 43 classes, 104 orders, 188 families, 391 genera and 556 species of bacteria at all seven developmental stages of *I. persulcatus.* Proteobacteria was the dominant phylum, and *Rickettsia* was the dominant genus. We also annotated six orders, 28 families, 72 genera, and 158 species of virus. Among them, 80 species of four genera were found in three samples, and the second-generation adult ticks annotated the highest number of virus species. To the best of our knowledge, this is the first study to comprehensively analyze the microbial community composition of *I. persulcatus* at different developmental stages and annotated *R. japonica*, BVDV, and ASFV in *I. persulcatus*.

## Figures and Tables

**Table 1:** The number of sequences, bases, and the average length of different samples.

Sample	Sequences	Bases (bp)	Average Legnth (bp)
First-generation female adult ticks	193,013	79,599,688	412.41
Eggs	77,459	31,356,590	404.82
Larval ticks	691,824	287,258,716	415.22
Engorged larval ticks	141,628	57,750,469	407.76
Nymphal ticks	78,104	32,874,931	420.91
Engorged nymphal ticks	54,276	23,220,668	427.83
Second-generation adult ticks	93,433	39,060,954	418.06
Total	1,329,737	551,122,016	414.46

**Table 2:** Community richness and alpha diversity indices of different samples.

Sample	97%				
	Good’s Coverage (%)	Shannon	Simpson	Chao	ACE
First-generation female adult ticks	99.9754	1.70	0.2649	203	219
Eggs	99.9841	0.81	0.5598	146	149
Larval ticks	99.9963	1.98	0.2491	190	193
Engorged larval ticks	99.9888	2.25	0.2436	569	571
Nymphal ticks	99.9679	1.98	0.2136	128	123
Engorged nymphal ticks	99.9554	0.76	0.7510	144	147
Second-generation adult ticks	99.9706	2.20	0.1577	160	163

**Table 3:** Distribution proportion of samples at different developmental stages at the phylum level.

Sample	Proteobacteria (%)	Actinobacteria (%)	Firmicutes (%)	Bacteroidetes (%)	Cyanobacteria (%)	Other (%)
First-generation female adult ticks	97.45	1.59	0.75	0.17	-	0.04
Eggs	98.87	0.43	0.42	0.15	0.07	0.06
Larval ticks	14.19	56.23	27.30	2.26	-	0.02
Engorged larval ticks	73.26	16.78	6.53	1.87	1.13	0.43
Nymphal ticks	97.22	1.01	0.51	1.23	-	0.03
Engorged nymphal ticks	99.01	0.08	0.51	0.27	-	0.13
Second-generation adult ticks	87.91	1.44	7.24	3.22	-	0.19
Total proportion	81.13	11.08	6.18	1.31	0.17	0.13

**Table 4:** Distribution proportion of 30 bacterial genera of samples at different developmental stages.

Genus	Relative Abundance (%)							
	First-Generation Female Adult Ticks	Eggs	Larval Ticks	Engorged Larval Ticks	Nymph Ticks	Engorged Nymph Ticks	Second-Generation Adult Ticks	Total Proportion
Rickettsia	38.708	69.596	2.714	3.827	30.166	3.143	15.885	27.720
Candidatus *Lariskella*	26.870	27.503	1.036	34.363	0.028	0.034	25.380	16.459
*Pseudomonas*	3.245	0.393	0.767	0.259	0.459	88.883	13.513	15.360
*Enterobacteriaceae unclassified*	0.546	0.263	0.006	0.042	33.252	0.170	20.950	7.890
*Brevibacterium*	0.304	-	43.261	9.384	0.038	0.011	0.030	7.575
*Stenotrophomonas*	21.345	0.200	0.087	0.136	4.198	1.138	6.662	4.827
*Staphylococcus*	0.367	0.028	26.229	1.123	0.200	0.085	0.004	4.005
*Acinetobacter*	0.049	-	0.002	0.189	10.03	0.062	0.677	1.573
*Serratia*	0.017	-	-	0.017	10.835	0.049	0.028	1.564
*Dietzia*	0.223	-	7.700	2.935	-	-	0.049	1.558
*Brucella*	1.487	0.006	5.139	1.111	0.085	0.066	1.491	1.341
*Achromobacter*	4.432	-	0.113	0.0347	0.023	0.837	2.364	1.115
*Lysinibacillus*	0.104	-	-	0.282	0.011	0.038	7.161	1.085
*Delftia*	0.057	0.017	0.202	0.074	4.279	2.801	0.057	1.070
*Brevundimonas*	0.093	0.013	1.368	0.890	3.321	0.357	0.342	0.912
*Brachybacterium*	0.062	-	3.544	0.52	0.454	0.004	0.015	0.657
*Sphingobacterium*	0.087	-	2.183	0.242	0.750	0.042	0.973	0.611
*Rhodococcus*	0.741	0.021	0.083	1.213	0.189	0.036	1.138	0.489
*Advenella*	0.040	-	2.164	0.106	0.011	-	0.002	0.332
*Jeotgalicoccus*	0.015	-	0.66	1.567	0.055	-	-	0.328
*Chryseobacterium*	0.002	0.002	-	0.028	0.021	0.019	2.034	0.301
*Corynebacterium*	-	-	0.032	1.867	0.181	0.006	-	0.298
*Microbacterium*	0.036	-	1.255	0.070	-	-	0.047	0.201
*Chloroplast_norank*	-	0.021	-	1.075	-	-	-	0.157
*EscherichiaShigella*	-	0.567	0.004	0.053	0.002	0.023	-	0.093
*Flavobacterium*	-	0.002	-	0.0539	-	0.002	0.104	0.093
*Psychrobacter*	0.079	-	-	0.539	-	-	0.017	0.091
*Comamonas*	0.002	-	-	-	0.089	0.459	0.066	0.088
*Bacillus*	0.104	0.117	0.053	0.183	0.006	0.127	0.011	0.086
*Bacteroides*	0.004	0.053	0.002	0.406	0.064	0.021	0.015	0.081

**Table 5:** Relative abundance of bacterial species of samples at different developmental stages.

Bacterial Species	Relative Abundance (%)							
	First-Generation Female Adult Ticks	Eggs	Larval Ticks	Engorged Larval Ticks	Nymph Ticks	Engorged Nymph Ticks	Second-Generation Adult Ticks	Total Abundance
*Rickettsia japonica*	38.708	69.596	2.714	33.827	30.166	3.143	15.885	27.720
*Ixodes persulcatus* taiga tick	26.870	27.503	1.036	34.363	0.028	0.034	25.380	16.459
*Pseudomonas aeruginosa*	0.074	0	0.134	0.017	0.151	86.517	0.212	12.444
*Enterobacteriaceae* unclassified	0.546	0.263	0.006	0.042	33.252	0.170	20.950	7.890
*Brevibacterium epidermidis*	0.304	0	43.248	9.382	0.038	0.011	0.030	7.573
*Stenotrophomonas* sp. MYb57	20.338	0.025	0.068	0.036	2.440	0.087	6.464	4.208
*Staphylococcus lentus*	0.329	0.013	23.309	1.068	0.151	0.040	0	3.559
*Pseudomonas* unclassified	2.395	0.316	0.626	0.106	0.282	2.130	11.803	2.523
*Serratia marcescens*	0	0	0	0.017	10.826	0.049	0.015	1.558
*Brucella* unclassified	1.487	0.006	5.139	1.111	0.085	0.066	1.491	1.341
*Acinetobacter* unclassified	0.002	0	0.002	0.181	8.807	0.051	0.093	1.305
*Achromobacter* unclassified	4.432	0	0.113	0.034	0.023	0.837	2.364	1.115
*Delftia tsuruhatensis*	0.057	0.017	0.202	0.074	4.279	2.801	0.057	1.070
Lysinibacillus_unclassified	0.104	0	0	0.002	0.011	0.038	7.161	1.045

**Table 6:** Biological classification of samples at different developmental stages.

Sample	Kingdom	Phylum	Class	Order	Family	Genus	Species
First-generation adult ticks	norank	norank	norank	6	27	64	136
Mixed sample	norank	norank	norank	5	23	51	90
Second-generation adult ticks	norank	norank	norank	5	27	71	143
Total	-	-	-	6	28	72	158

## Data Availability

The raw tags have been deposited in Sequence Read Archive (SRA) from the NCBI under BioProject accession number PRJNA1174404 and PRJNA1181586. The individual run files received the accession numbers SAMN44349064–SAMN44349070 and SAMN44564509–SAMN44564511.
